# *Dubinectes infirmus*, a new species of deep-water Munnopsidae (Crustacea, Isopoda, Asellota) from the Argentine Basin, South Atlantic Ocean

**DOI:** 10.3897/zookeys.144.1578

**Published:** 2011-11-03

**Authors:** Marina Malyutina, Angelika Brandt

**Affiliations:** 1 A.V. Zhirmunsky Institute of Marine Biology, FEB RAS, Palchevskogo 17, 690059; Far East Federal University, Sukhanova Str., 8, 690950, Vladivostok, Russia; 2 Biozentrum Grindel und Zoologisches Museum, Martin-Luther-King-Platz 3, 20146 Hamburg, Germany

**Keywords:** deep sea, South Atlantic, taxonomy, Munnopsidae, *Dubinectes*, new species

## Abstract

*Dubinectes infirmus* **sp. n.**, Munnopsidae, is described from the Argentine Basin, southwest Atlantic, at depths between 4586–4607 m. The new species is distinguished by a narrow rim of the pleotelson posterior margin which is not raising over its dorsal surface; article 3 of the antennula is subequal in length to article 2; distomedial lobes of male pleopod 1 are of same size as distolateral lobes; stylet of male pleopod 2 is subequal in length to protopod; uropod exopod is more than a half of endopod length. Some generic characters which are weakly pronounced in the new species or have different state are defined more precisely in the revised diagnosis of *Dubinectes*. The modified diagnosis of the genus, a key to the species of *Dubinectes* as well as the distribution of the genus are presented.

## Introduction

Isopods of the family Munnopsidae were collected in 2009 during the DIVA 3 (Diversity of the Abyssal Atlantic) expeditions onboard RV *Meteor* in 2009 in the southwest Atlantic (M 79-1). Some known, rare munnopsid species originally described from ANDEEP (Antarctic benthic DEEP-sea biodiversity: colonization history and recent community patterns) collections, e.g. species of *Microcope* Malyutina, 2008 and *Dubinectes* Malyutina & Brandt, 2006, were identified in the DIVA 3 samples taken from the Argentine Basin. The genus *Dubinectes* was erected for four species from the southeast Atlantic and Weddell Sea: *Dubinectes nodosus* (Menzies, 1962) and *Dubinectes acutitelson* (Menzies, 1962), which were formerly placed in *Eurycope* Sars, 1864 and *Dubinectes intermedius* Malyutina & Brandt, 2006 and *Dubinectes acutirostrum* Malyutina & Brandt, 2006. Along with the known species, *Dubinectes intermedius* and *Dubinectes nodosus* a new species of *Dubinectes* was found in the DIVA 3 samples*.* Though the new species possesses the main diagnostic characters of the genus, some of these characters have different state in contrast to other species of *Dubinectes* and this allows to define more precisely the generic diagnosis. In this paper we describe a new species, the fifth species of the genus, present the revised diagnosis of *Dubinectes*, a key of the genus, new records of two known species, as well as the distribution of the genus.

## Material and methods

Specimens were collected using an epibenthic sledge (Brenke 2005) and then fixed in pre-cooled 96% ethanol. The material was sorted in the laboratory and identified using a Leica MZ 12 stereomicroscope equipped with a camera lucida, and drawn using an Olympus SZX7 compound microscope, also equipped with a camera lucida.

The terminology and measurements mostly follow Wilson (1989). Total body length was measured medially from the tip of the rostrum to the posterior tip of the pleotelson. The dorsal view was used for measuring the width, while the length of body segments was measured in lateral view. For the description of the body and pereopods the holotype was used, for mouthparts and pleopods a male paratype was dissected and for some details that the paratype lacked or were different from male, a female paratype was used. Scale bars for total body figures are 1 mm.

## Abbreviations

ZMH – Zoological Museum of Hamburg, An1 – antennula, An2 – antenna 2, lMd – left mandible, rMd – right mandible, Mx1 – maxilla 1, Mx2 – maxilla 2, Mxp – maxilliped, P1–7 – pereopods 1–7, bP – basis of pereopod, Plp 1–5 – pleopods 1–5, Ur – uropod.

## Taxonomy

**Family Munnopsidae Lilljeborg, 1864**

**Subfamily Eurycopinae Hansen, 1916**

### Genus Dubinectes

Malyutina & Brandt, 2006

http://species-id.net/wiki/Dubinectes

Dubinectes Malyutina & Brandt, 2006: 4.

#### Composition.

*Dubinectes acutitelson* (Menzies, 1962) type species; *Dubinectes nodosus* (Menzies, 1962); *Dubinectes acutirostrum* Malyutina & Brandt, 2006; *Dubinectes intermedius* Malyutina & Brandt, 2006; *Dubinectes infirmus* sp. n.

**Figure 1.**
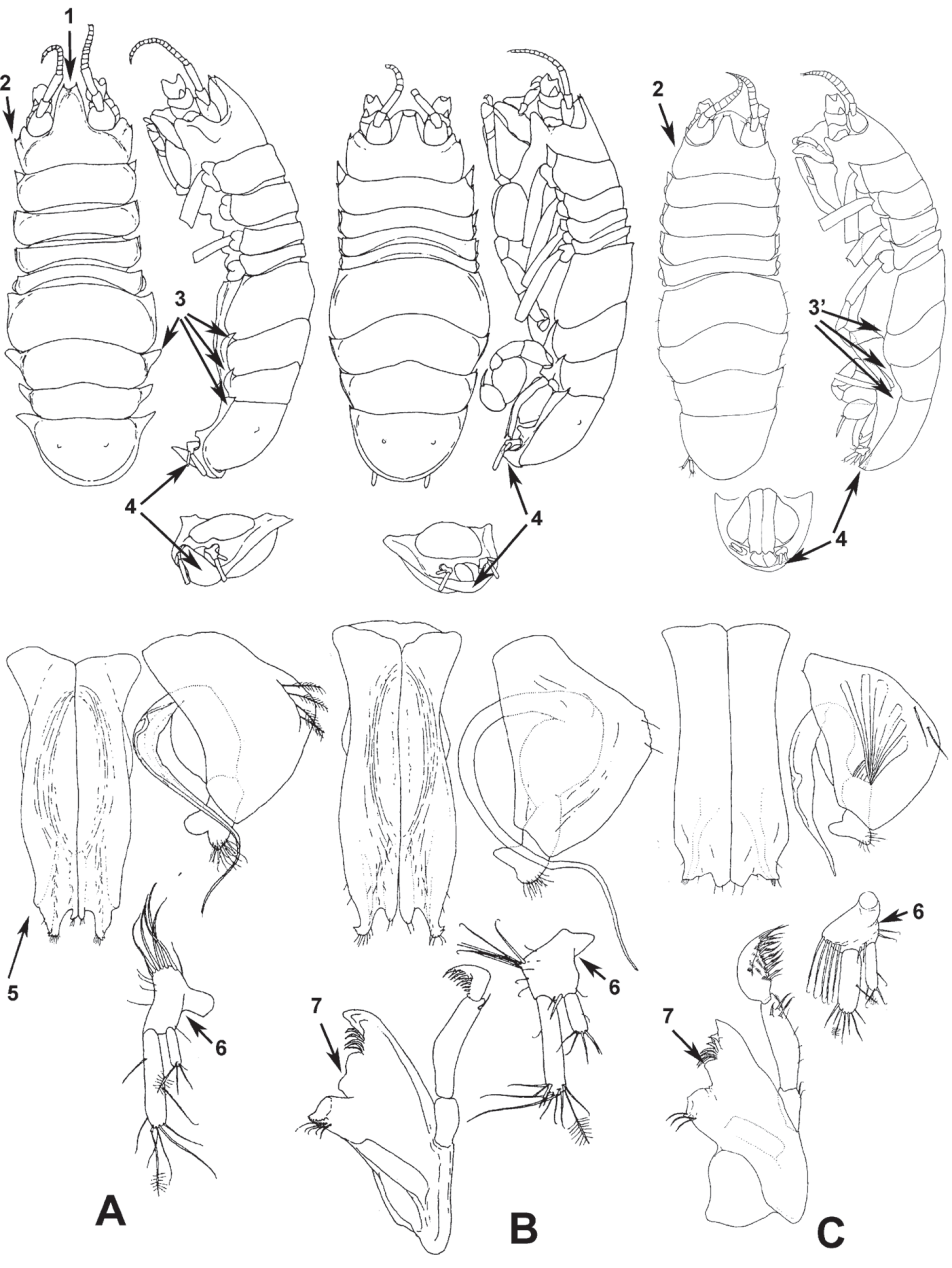
Illustrations of the specific characters of the genus *Dubinectes* used in the Key and the Diagnosis. Vertical rows **A, B**, and **C** for each species include total views, male pleopods 1, 2, uropod and mandible: **A** *Dubinectes acutitelson* **B** *Dubinectes nodosum*, and **C–D***. infirmus* sp. n.: **1** distal notch of rostrum **2** ledge of head anterolateral margin; **3** projected, dorsally raised anterolateral margins of pereonites 6, 7 and pleotelson **3’** weakly pronounced, not dorsally raised anterolateral margins of pereonites 6, 7 and pleotelson **4** posterior rim of pleotelson with vental process (**A**) and without process (**B** and **C**) **5** ledge of pleopod 1 distolateral margin **6** concave lateral inflexion of uropod protopod **7** ridge between spine row and molar of mandible.

Key to the species of *Dubinectes* (see Fig. 1).

**Table:** 

1	Cuticle of body strongly calcified; rostrum robust, about twice as long as antennula article 1; anterolateral margins of pereonites 6, 7, pleotelson and posterior rim of pleotelson projected, raised dorsally	2
–	Cuticle of body weakly calcified; rostrum only slightly longer than antennula article 1; anterolateral margins of pereonites 6, 7, pleotelson and posterior rim of pleotelson weakly pronounced, not raised dorsally	3
2	Rostrum with distal notch, ledge on anterolateral margins of head smooth; lateral margins of pleopod 1 smooth	*Dubinectes acutitelson* (Menzies, 1962)
–	Rostrum pointed, ledge on anterolateral margins of head rectangular acute; pleopod 1 with small distolateral ledge	*Dubinectes acutirostrum* Malyutina & Brandt, 2006
3	Posterior rim of pleotelson not raising dorsally, rostrum tip rounded; antennula article 3 subequal or shorter than article 2; distolateral and distomedial lobes of male pleopod 1 subequal	*Dubinectes infirmus* sp. n.
–	Posterior rim of pleotelson raising dorsally, rostrum with distal notch; antennula article 3 obviously longer than article 2; distolateral lobes of male pleopod 1 longer than distomedial lobes	4
4	posterior rim of pleotelson with rounded posteroventral process	*Dubinectes intermedius* Malyutina & Brandt, 2006
–	posterior rim of pleotelson narrow, ventral process almost absent	*Dubinectes nodosus* (Menzies, 1962)

#### Modified generic diagnosis.

Pleotelson posterior margin surrounded with rim, running perpendicular to margin. Uropod protopod bent at midlength, both margins angled: concave lateral inflexion and projected medial corner, bearing long setae. Body deepest at head. Rostrum of head longer than antennula article 1, with 2 robust distal setae. Distomedial lobe of antennula article 1 reduced, shorter than distolateral projection. Head and pereonites 1–4 subequal in width, pereonite 1 longest, pereonite 4 nearly third of length of pereonite 1, pereonite 7 not longer than pereonite 6. Mandible with high sharp ridge between short spine row and broad molar; condyle longer than molar. Pereopods 1–4 subequal in length. Male pleopod 1 distolateral and distomedial lobes well separated from each other by broad notch, distolateral lobes produced, not narrower than distomedial lobes. Male pleopod 2 protopod distal margin truncated; exopod inserting close to distal margin and emerging posteriorly; endopod basal article twice as broad as stylet; stylet subequal or longer than protopod.

#### Remarks.

Before finding the *Dubinectes infirmus* sp. n.the key characters which helped to easily determine known species of *Dubinectes* without dissection were the strongly calcified integument of the body and the special shape of the pleotelson rim which is well projected ventrally and rises over the dorsal surface. These external features are only weakly pronounced in the new species.Nevertheless, it possesses these characters as well as the main set of diagnostic characters of *Dubinectes*. The new species has a rim of a pleotelson even if it is narrow, a rostrum which is longer than antennula article 1, a reduced distomedial lobe of the antennula article 1. The head and the ambulosome of the new species are of equal width, the pereonites are progressively shorter from 1 to 4; the coxa of pereopod 4 is broadest in lateral view; the mandible possesses a high sharp ridge between a short spine row and a broad molar; the condyle is longer than the molar; the distal margin of the male pleopod 2 protopod is truncated and the exopod is inserted close to the distal margin and emerged posteriorly. There are some characters which differ the new species from others. These are the shape of the distal margin of male pleopod 1, the length of the stylet of male pleopod 2, the length of the antennula article 3 as well as the length of uropod exopod. These characters in different states were suggested earlier as generic, but seem to bejust specific characters. Therefore, the description of *Dubinectes infirmus* sp. n. allows to revise the diagnosis of the genus.

#### Distribution of the genus *Dubinectes* (Fig.10).

The genus *Dubinectes* occurs in the Southern hemisphere only in the South Atlantic Ocean. Species of the genus were known from the southeast Atlantic and the Weddell Sea, Southern Ocean, from depths ranging between 1121 and 4960 m. New records the DIVA 3 expedition from the abyss of the Argentine Basin expand the geographical range for the genus to the southwest Atlantic until 27° S latitude in the north. Geographically, *Dubinectes nodosus* is the most widely distributed species of the genus. It is most eurybathic and has been sampled between 1121 to 4976 m. It was collected in the Weddell Sea at almost every station during the ANDEEP expeditions (Malyutina and Brandt 2007), but it was much less abundant in the DIVA collections. All other species were recorded only from abyssal depths between 4586 to 4976 m. *Dubinectes acutirostrum* and *Dubinectes infirmus* sp. n. are known only from their type localities.

### Dubinectes infirmus sp. n.

urn:lsid:zoobank.org:act:86B0A7BB-DEDF-45CF-9CE6-A7E19C29BAAB

http://species-id.net/wiki/Dubinectes_infirmus

**Figure 2.**
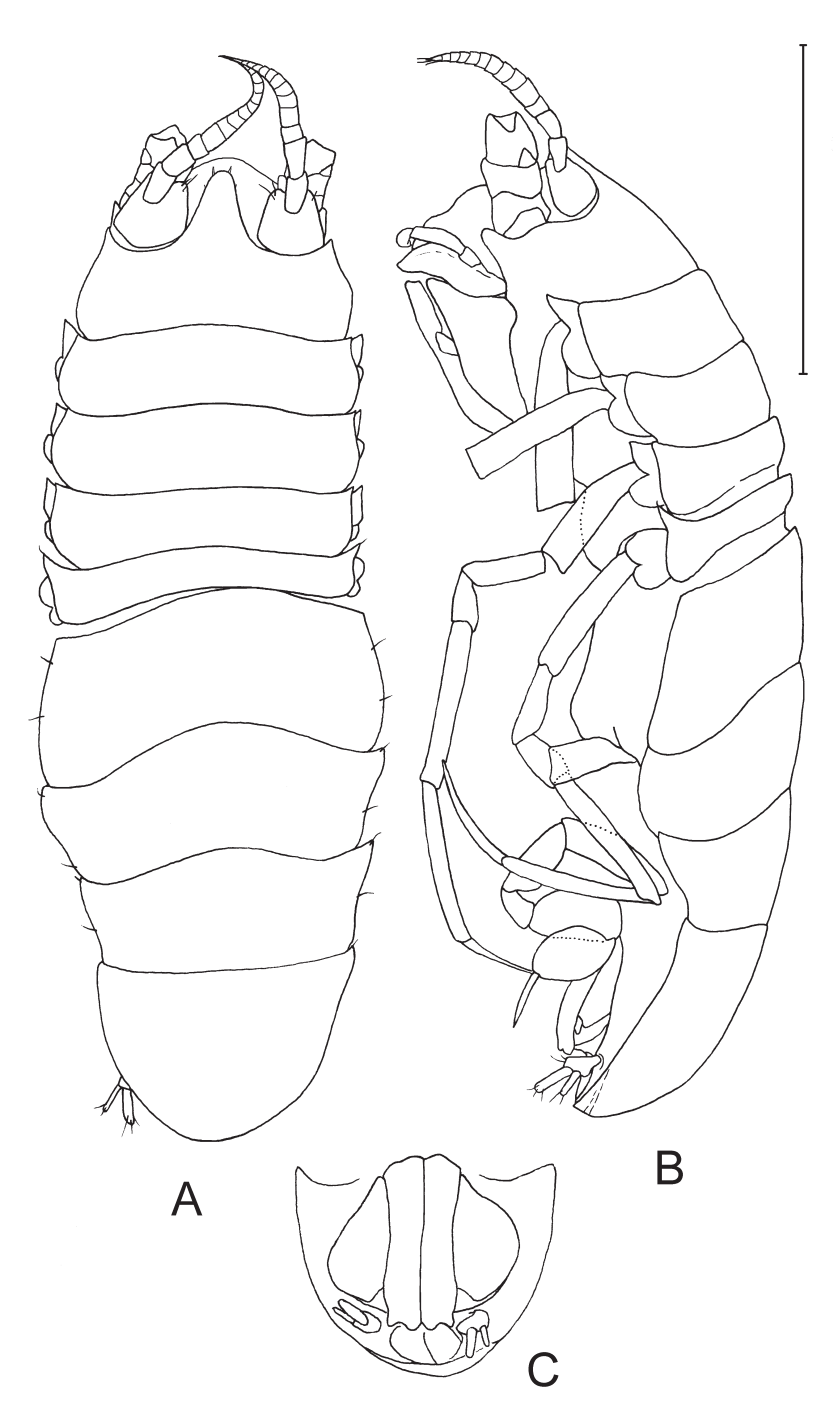
*Dubinectes infirmus* sp. n.Holotype male (ZMH 42969) **A** body, dorsal view **B** body, lateral view **C** pleotelson, ventral view.

Figs 2 [Fig F3] [Fig F4] [Fig F5] [Fig F6] [Fig F7] [Fig F8] –9 

#### Material examined.

Argentine Basin, RV *Meteor*.

*Holotype:*(ZMH 42969), male (3.0 mm), DIVA 3 Station 534 16.07.2009 36°00.61'S, 49°01.55'W, 4586-4605 m.

*Paratypes:*(ZMH 42970) damaged male used for dissection of mouthparts and pleopods, female 3.0 mm, male 3.1 mm, female 3.8 mm, mancas 2.5 and 2.1 mm, the same sample from the same locality; (ZMH 42971) 1 male, 2 females and 2 mancas (1.8-3.4 mm), Station 532, 15.07.2009 35°59.24'S, 49°00.86'W, 4605-4607 m.

#### Diagnosis.

Rostrum of head with rounded apex; antennula article 3 subequal in length to article 2; anterolateral margins of pereonites 6, 7, pleotelson and posterior rim of pleotelson weakly pronounced, not raised dorsally; pleopod 1 distomedial lobes of the same size as distolateral lobes; endopod stylet of male pleopod 2 subequal in length to protopod. Uropod exopod more than half of endopod length.

#### Description.

**Figure 3. F3:**
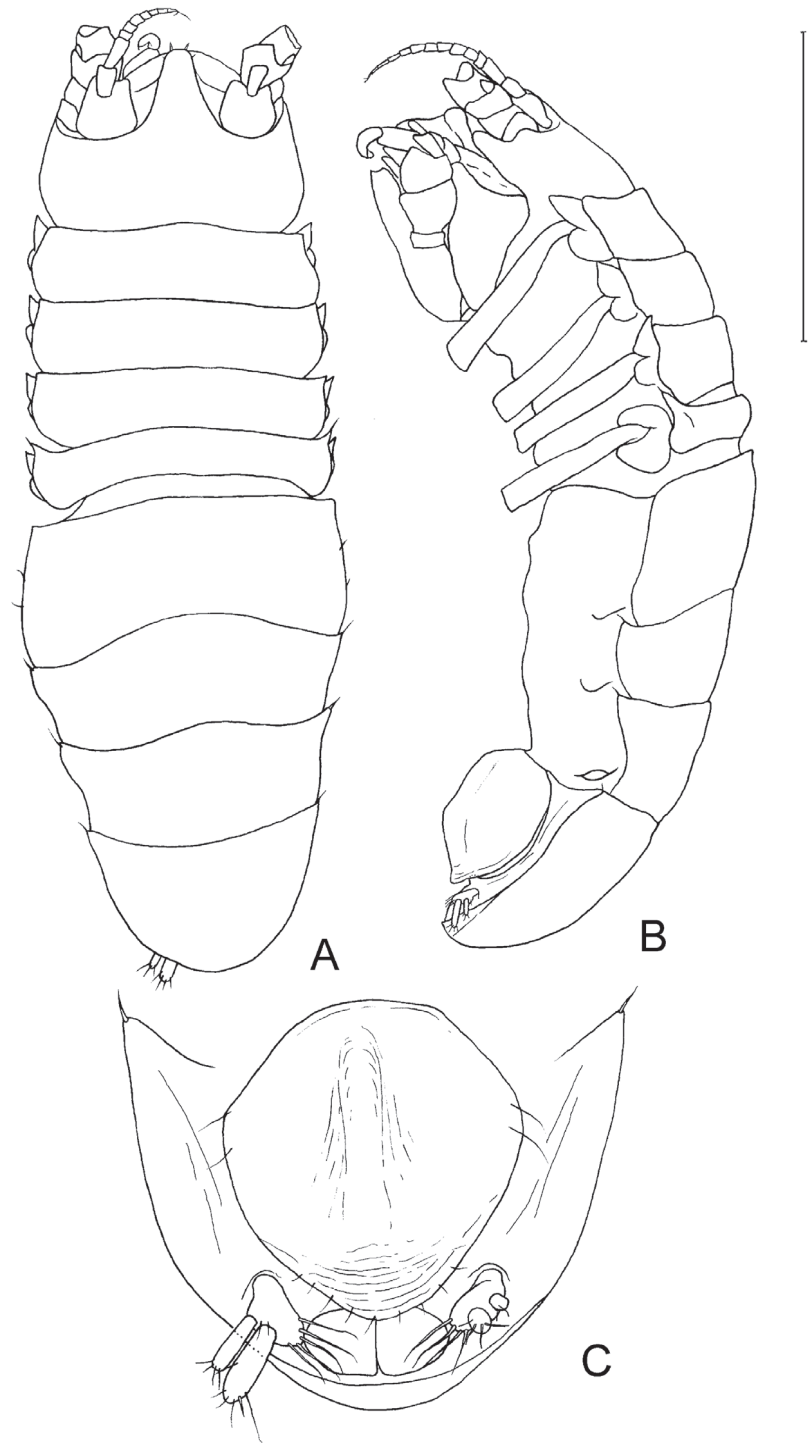
*Dubinectes infirmus* sp. n**.** Paratype female (ZMH 42970) **A** body, dorsal view **B** body, lateral view **C** pleotelson, ventral view.

Holotype male: body (Fig. 2) length 2.8 times width of pereonite 5, body height 0.25 of body length. Head length 0.55 times width; length behind antennula insertion 1.1 antennula article 1 length; rostrum as long as proximal part of head, distally convex, with 4 setae; clypeus 1.6 as wide and 0.5 as long as labrum. Pereonite 1 almost as wide as head, length 0.8 length of head behind antennula insertion; pereonites 1–4 subequal in width, shortening from 1–4, pereonite 4length about half of pereonite 1 length; anterolateral margins of pereonites 1–4 and anterior projections of coxae acute, anterolateral angles of pereonite 4 slightly projected, terminating with seta. Natasome length 0.5 body length, width 1.3 width of head; pereonite 5 longest, lateral length 0.7 of lateral length of pereonites 6 and 7 together; pereonite 7 shortest, 0.8 times length of pereonite 5. Pleotelson length 0.9 width, slightly longer than head, 0.25 body length, preanal ventral ridge weakly projected, rounded terminally; anterolateral corners of pereonites 6, 7 and pleotelson acute, weakly projected, terminating with seta. Proportions of habitus and body parts in females and juveniles (Figs 3, 8) are equal to those of the holotype.

**Figure 4. F4:**
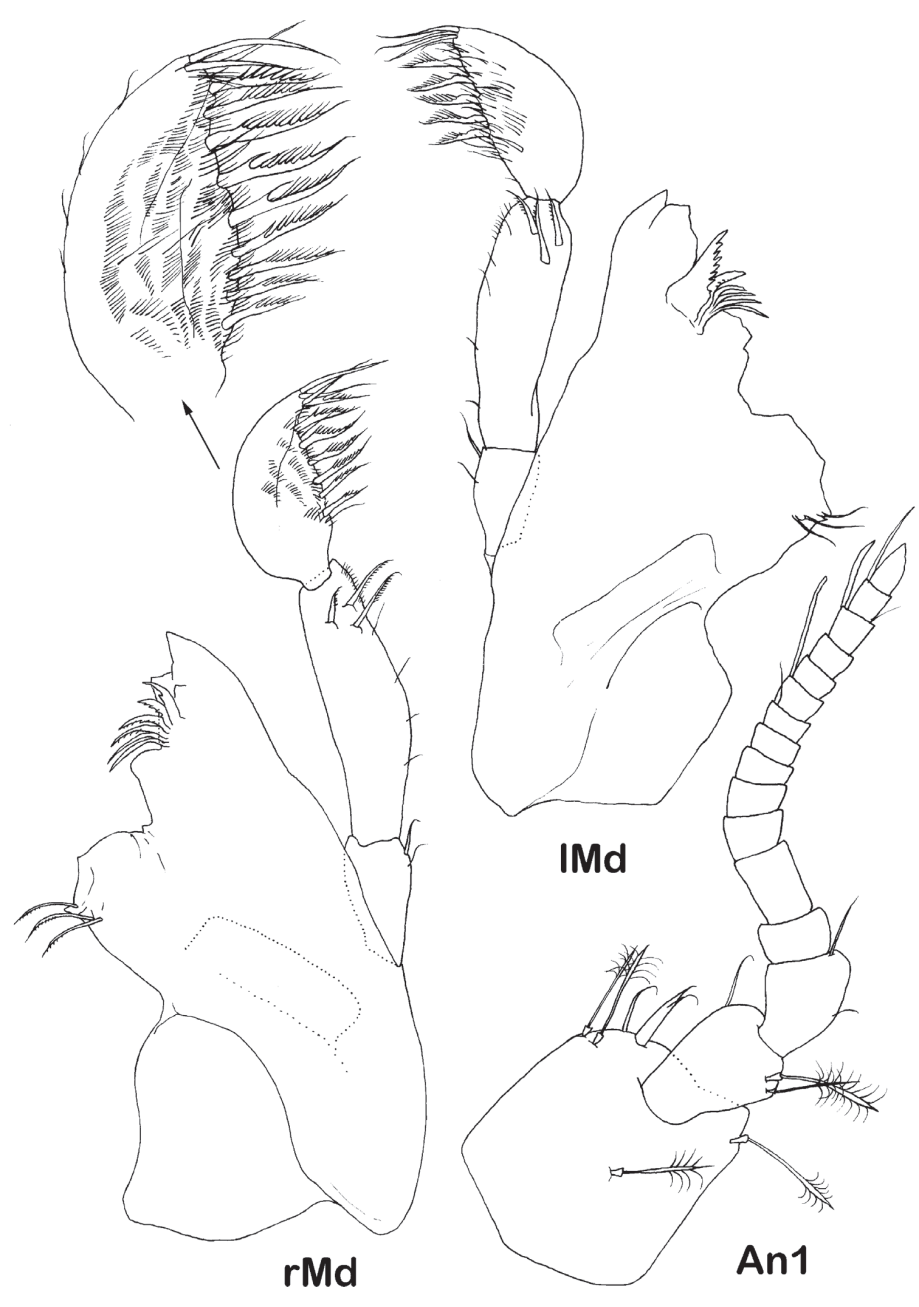
*Dubinectes infirmus* sp. n.Paratype male (ZMH 42970). Antennula and mandibles, right mandible with detail of distal palp article.

Antenna 1 of male (Fig. 4) article 1 1.05 times width, setation: 1 dorsal and 1 distolateral broom seta and 1 simple seta, 1 stout unequally bifid and 2 broom setae distomedially; article 2 0.6 times length and 0.4 width of article 1, with 1 simple and 2 distal broom setae; article 3 0.7 length of article 2 with 2 simple setae, article 4 0.3 times length of article 3, article 5 twice as long as article 4, following 11 flagellar articles subequal in length to article 4, some of the distal articles with an aesthetasc. Antenna 1 of female (Fig. 9) article 2 length 0.65 times length of article 1, article 3 slightly longer (1.1) than article 1, article 4 length 0.35 times article 3 with broom seta and aesthetasc distally; flagellum of 5 articles, last one with 1 aesthetasc and 5 setae.

Antenna 2 of female(Fig. 8) articles 1–4 subequal in size, length together 0.6 times body length, scale on article 3 nearly half the length of article 4. Article 5 almost as wide and 2.1 times as long as articles 1–4 together, with 3 stout medial setae and 2 small simple distolateral setae, article 6 narrower and 1.1 times as long as article 5, with simple setae along article. Flagellum broken off, articles subequal in length to article 1.

Mandibles (Fig. 4) longitudinal outer keel of body weakly pronounced, incisor process with 2 acute and 1 weak cusps; *lacinia mobilis* of left mandible triangular with many denticles, 0.8 times length of incisor process; spine row with 5 and 6 spines on left and right mandibles respectively; molar process 0.5 times as long and as broad as incisor process on proximal part, ventral margin of triturative surface with a few denticles and 3 setulose setae; ridge between spine row and molar with 1 pick; condyle 0.3 times body length, longer than molar; palp 0.9 times body length; article 1 with 2 distal setae, article 2 broadened at midlength, length 2.2 times article 1 length, with 3 stout distal setae and small marginal setae, article 3 0.75 of length and 1.15 of width of article 2, with 3 long simple distal setae and long comb-like marginal setae.

**Figure 5. F5:**
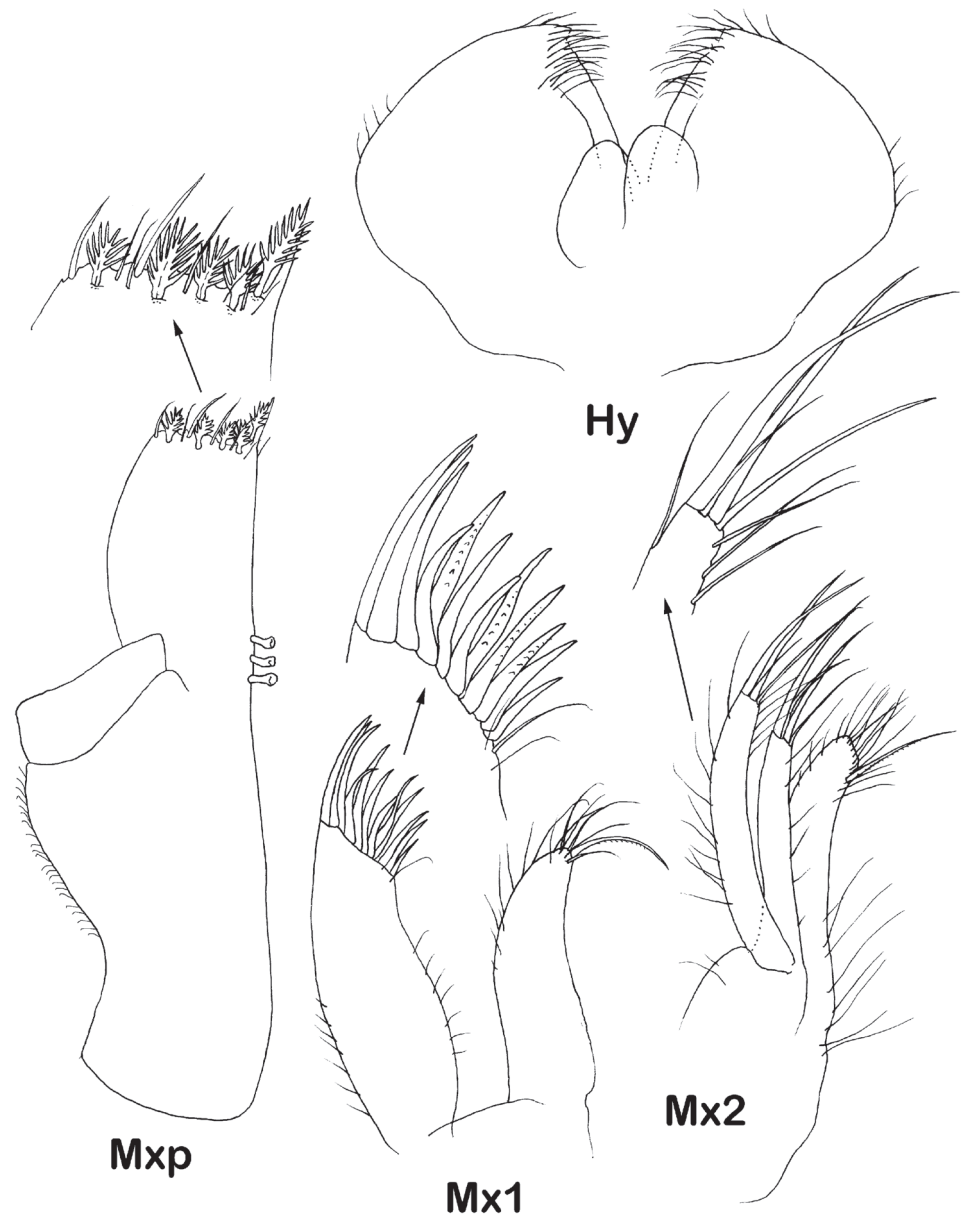
*Dubinectes infirmus* sp. n.Paratype male (ZMH 42970). maxilliped, with detail of endite distal margin, left maxilla 1 with detail of distal margin of lateral lobe, left maxilla 2 with detail of distal margin of lateral lobe, hypopharynx.

Hypopharynx (Fig. 5) outer lobes 3.7 times as wide as inner lobes with dense distal fine setae and four lateral setae each.

Maxilla 1 (Fig. 5) lateral endite 1.35 times as wide as mesial endite, with 12 distal stout serrated setae, mesial endite distally with tuft of small slender setae and 1 long setulate seta.

Maxilla 2 (Fig. 5) middle endite shortest, lateral endite longest, mesial endite with dense tuft of distal setae, longest one setulate; lateral and mesial endites each with 2 long and 2 shorter distal setae.

Maxilliped (Figs 5, 9) basis 2.7 times width, endite with 3 coupling hooks (2 in immature female), distal margin with 4-5 large fan setae and numerous simple slender setae; palp article 2 margins almost straight, lateral margin 1.3 times longer than medial margin; article 3 medial margin slightly convex, with low denticles and 5 setulose setae, 1.2 times longer than medial margin of article 2; article 4 2 times longer than articles 3 laterally, medial lobe almost not produced, with 4 distal setae, article 5 0.9 of article 3 laterally, with 7 distal setae. Epipod subequal in length to basis length 1.6 times width, lateral extension rounded, almost as broad as distal angle.

Pereopods 1–4 bases subequal in size. Pereopod 1 (Fig. 9) 0.3 times body length, length ratios of ischium-propodus to basis: 0.5, 0.3, 0.9, 0.7, 0.2; basis with small setae: 3 ventral and 1 dorsal; merus with 1 small ventral seta; carpus with 4 small ventral and 3 dorsal setae; propodus only slightly narrower than carpus, with 3 ventral and 5 dorsal setae; dactylus with elongate claw, 3 slender setae proximally claw.

**Figure 6. F6:**
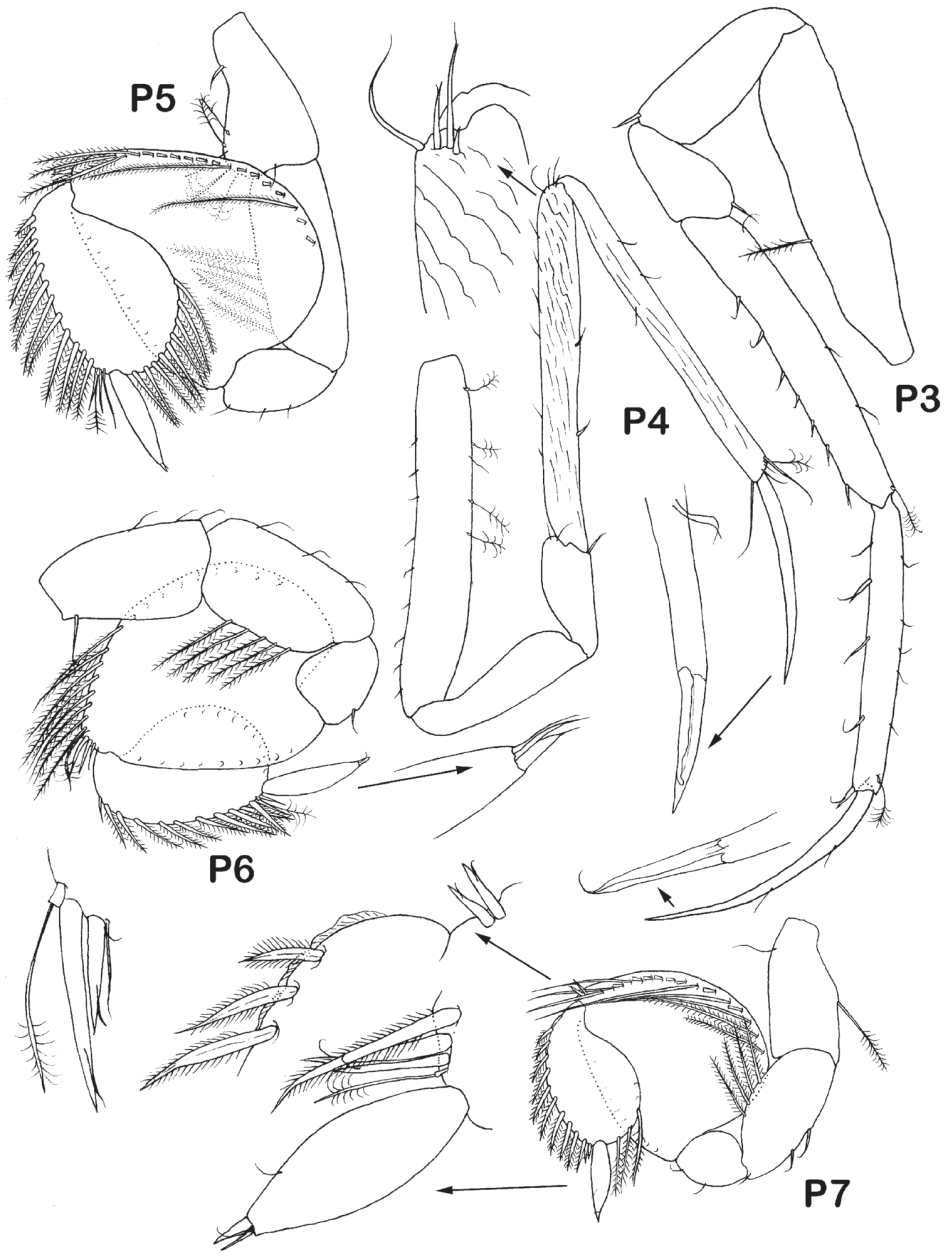
*Dubinectes infirmus* sp. n.Holotype male (ZMH 42969). Pereopods 3-7 with enlarged details.

Pereopod 3 (Fig. 6) length ratios of ischium-propodus to basis: 0.5, 0.3, 1.0, 0.9, 0.75; basis with 1 plumose dorsal seta, ischium with 1 distoventral seta, merus with 1 distodorsal seta, carpus with 7 short unequally bifid ventral setae, 1 broom distodorsal seta and 3 small dorsal setae, propodus 0.7 of carpus width, with 5 unequally bifid ventral setae longer than these on carpus, 1 distodorsal seta and 5 small dorsal setae, dactylus with elongated claw. Pereopod 4 (Fig. 6) slightly shorter and more slender than pereopod 3, propodus longer and dactylus shorter than these in pereopod 3; length ratios of ischium-propodus to basis: 0.5, 0.3, 1.05, 1.05, 0.6; basis with 4 plumose dorsal and 9 simple small ventral setae; ischium without setae, merus with 3 distal setae; carpus with 2 short unequally bifid ventral setae, 1 whip and 3 stout unequally bifid distodorsal setae and 6 small dorsal setae; propodus 0.6 of carpus width, with 1 ventral and 2 dorsal small simple setae, and1 broom seta, 2 whip and 2 small setae distally.

Pereopods 5–7 (Figs 6, 9) of same shape, decreasing from 5 to 7, with stout basis- ischium, bases lengths about half of bases of pereopods 1*–*4; plumose marginal setae on carpi elongate, longest setae length about 0.7 of carpus width; plumose marginal setae on propodi stout, about half length of marginal setae on carpi; both articles with the same set of short setae distodorsally: 2 stout unequally bifid, 1 broom and 1 whip seta; dactyli with tiny claw and 2 setae distoventrally. Pereopod 5 length 0.65 pereopod 3 length; length ratios of ischium-carpus to basis: 1.3, 0.7, 1.6, 1.2, 0.6; basis with 5 dorsal broom setae; ischium with 5 dorsal plumose setae; merus with 3 small ventral simple setae; carpus length 1.3 times width, with 19 dorsal and 10 ventral plumose elongate setae; propodus 1.3 times width, with 12 dorsal and 9 ventral plumose stout setae; dactylus width 0.2 propodus width. Pereopod 6 length 0.9 pereopod 5 length; length ratios of ischium-dactylus to basis: 1.05, 0. 5, 1.5, 1.0, 0.6; basis with 1 dorsal and 3 ventral setae; ischium with 5 plumose dorsal and 3 simple ventral setae; merus with 1 ventral seta; carpus length 1.3 times width, with 17 dorsal and 9 ventral plumose elongate setae; propodus 1.7 times width, with 11 dorsal and 9 ventral stout plumose setae; dactylus width 0.2 propodus width. Pereopod 7 length 0.7 pereopod 5 length, length ratio of ischium-dactylus to basis: 1.05, 0.5, 1.3, 1.0, 0.6; basis with 1 plumose ventral seta and 1 whip dorsal seta; ischium with 4 plumose dorsal and 2 ventral setae; merus with 2 small ventral setae; carpus 1.05 times width, with 16 dorsal and 8 ventral elongate plumose setae; propodus 1.6 times width, with 10 dorsal and 5 ventral stout plumose setae; dactylus width 0.3 propodus width.

**Figure 7. F7:**
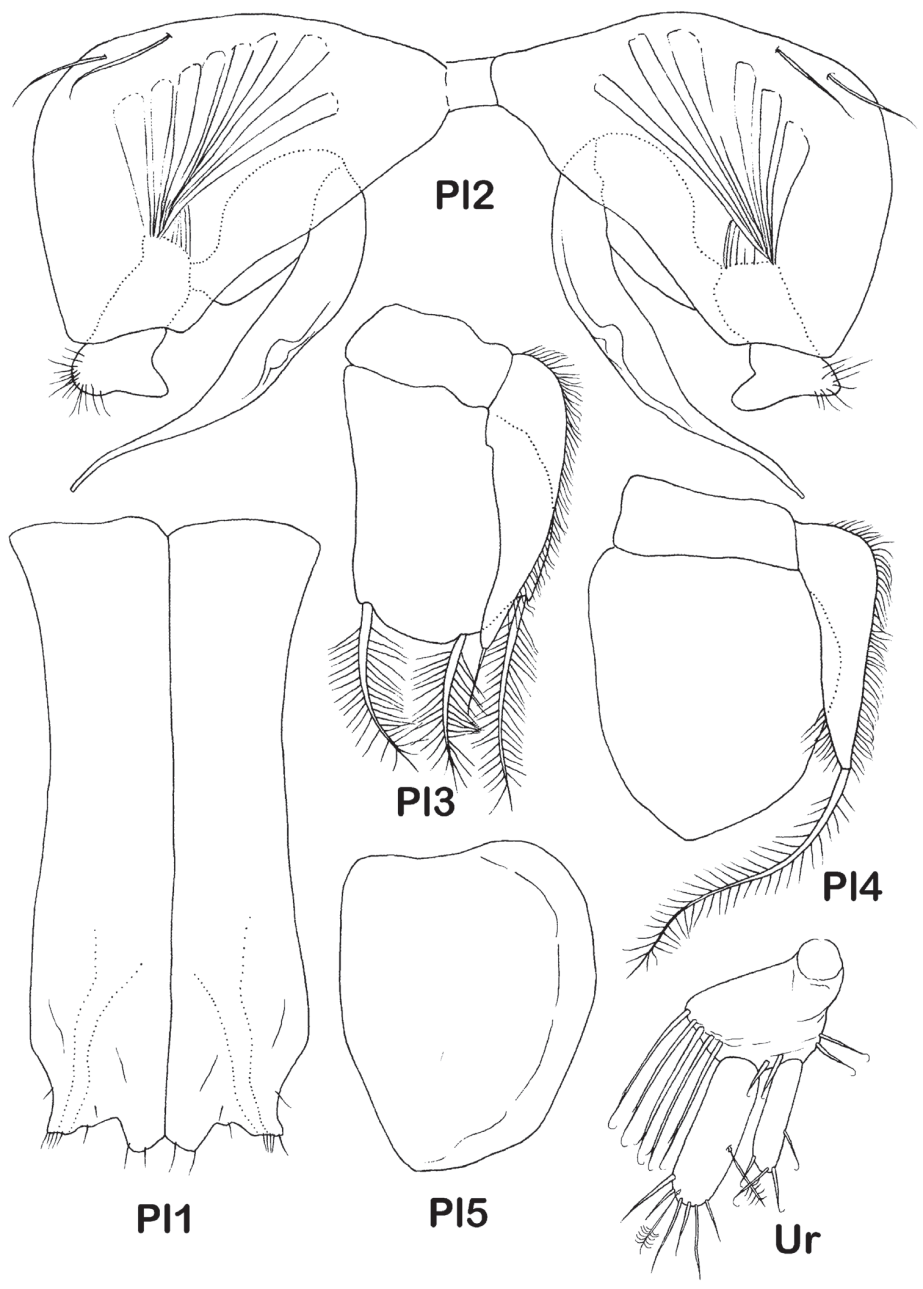
*Dubinectes infirmus* sp. n. Paratype male (ZMH 42970). Pleopods 1-5, right uropod.

Pleopods (Fig. 7): pleopod 1 of male 2.25 times width, distal margin: lateral lobes acute slightly projected laterally, with 5 distal small setae each; medial lobes rounded, almost as wide as and slightly longer than lateral lobes, with 2 small distal setae each. Pleopod 2 of male protopod length 1.7 times width, 0.75 as long and as wide as pleopod 1, with 2 lateral submarginal setae, stylet of endopod length 0.9 protopod length, exopod prominent and visible behind distal truncated margin of protopod, distolateral hook rounded, with tuft of fine setae. Pleopod 3 endopod 1.3 times width, 3 distal plumose setae only slightly shorter than endopod; exopod basal width 0.4 endopod width, length 1.1 endopod length, reaching beyond endopod distal margin, with dense row of hair-like lateral setae and 1 distal simple seta, length 0.25 exopod length. Pleopod 4 endopod length 1.3 times width, exopod 0.4 of width and 0.9 of length of endopod, with dense row of lateral slender setae, distal seta plumose, length 1.4 exopod length. Pleopod 5 endopod length 1.4 times width.

Pleopod 2of female as long as wide, with medial keel, 2 long simple setae proximolaterally and 10 small simple setae on posterior margin (Fig. 3).

Uropod (Fig. 7) length 0.3 times pleotelson length; protopod as long as wide, with 5 long whip setae on medial projection and several distal setae, endopod 0.33 times as wide and slightly longer than protopod, with 1 lateral and one distal broom and 6 whip setae; exopod length 0.7 times endopod length, with 4 setae distally.

#### Distribution.

Southwest Atlantic: northern Argentine Basin at depths between 4586–4605 m.

#### Etymology.

The epithet infirmus (Latin) refers to the state of the generic characters of the species, which are less obvious than in other species.

#### Remarks.

*Dubinectes infirmus* sp. n. differs from the four other species of the genus by weakly pronounced generic characters: a rim, surrounding the pleotelson posterior margin is narrow, not raising over the dorsal surface; lateral margins of the head are without an obvious ledge, anterolateral angles of pereonites 5–7 and pleotelson not produced dorsally. Article 3 of male antennula in the new species is shorter than article 2, in females and juveniles it is slightly longer than article 2 in contrast to other species which have article 3 nearly twice as long as article 2; pleopod 1 distolateral lobes are subequal in size to the distomedial lobes (in other species they are broader and longer); the endopod stylet of male pleopod 2 is subequal in length to the protopod (0.9) in contrast to state in other species which is approximately two times the length of the protopod; both endopod and exopod of the uropod are rather stout, shorter than in other species, the exopod is 0.7 times the endopod length (0.35 – 0.5 in other known species)*.* The new species is the most similar to *Dubinectes nodosus* in body shape and size of the rostrum, but the new species has a less calcified cuticle, a rounded tip of the rostrum, a narrow posterior rim of the pleotelson, not raising dorsally, shorter article 3 of antennula and another configuration of distal margin of pleopod 1 than in *Dubinectes nodosus*.

### Dubinectes intermedius

Malyutina & Brandt, 2006

http://species-id.net/wiki/Dubinectes_intermedius

Eurycope acutitelson Menzies, 1962: 143 (partim)
Dubinectes intermedius Malyutina & Brandt, 2006: 37, Figs 19–22.

#### Material examined.

DIVA 3 Station 534, 16.07.2009, 36°00.61'S, 49°01.55'W, 4586-4605 m - 6 males; Station. 532, 15.07.2009, 35°59.24'S, 49°00.86'W, 4605-4607 m - 1 male, 2 females and 2 mancas; Station 533-1, 15.07.2009, 36°00.20'S, 49°01.96'W, 4601.8 m - 1 female, 2 mancas.

#### Distribution.

Off Capetown: 41°03.5'S, 07°49'E, 4960 m; Weddell Sea: 64°59.20'S, 43°02.05'W, 4698 m.

### Dubinectes nodosus

(Menzies, 1962)

http://species-id.net/wiki/Dubinectes_nodosus

Eurycope nodosa Menzies, 1962: 145, Fig. 36 F–H.
Dubinectes nodosus Malyutina & Brandt, 2006: 27, Figs 12–18.

#### Material examined.

DIVA 3, Station 533-1, 15.07.2009, 36°00.20'S, 49°01.96'W, 4601.8 m - 2 males, 1 females, 1 manca; Station 561, 27.07.2009 26°34.80'S, 35°13.89'W, 4482-4489 m - 1 male.

#### Distribution.

Cape Basin: 41°08'S, 14°08'E36°34'S, 09°56'W, 4672-4885 m; 47°39.87'S, 04°15.79'W, 2923 m; Weddell Sea: 68°04'S, 38°47.75'W62°58'S, 51°31.61'W, 3053-4976 m; 65°20.17'S, 54°14.30'W, 1121 m; Argentine Basin: 36°00.20'S, 49°01.96'W, 4601.8 m; off Brazil 26°34.80'S, 35°13.89'W, 4482-4489 m.

**Figure 8 F8:**
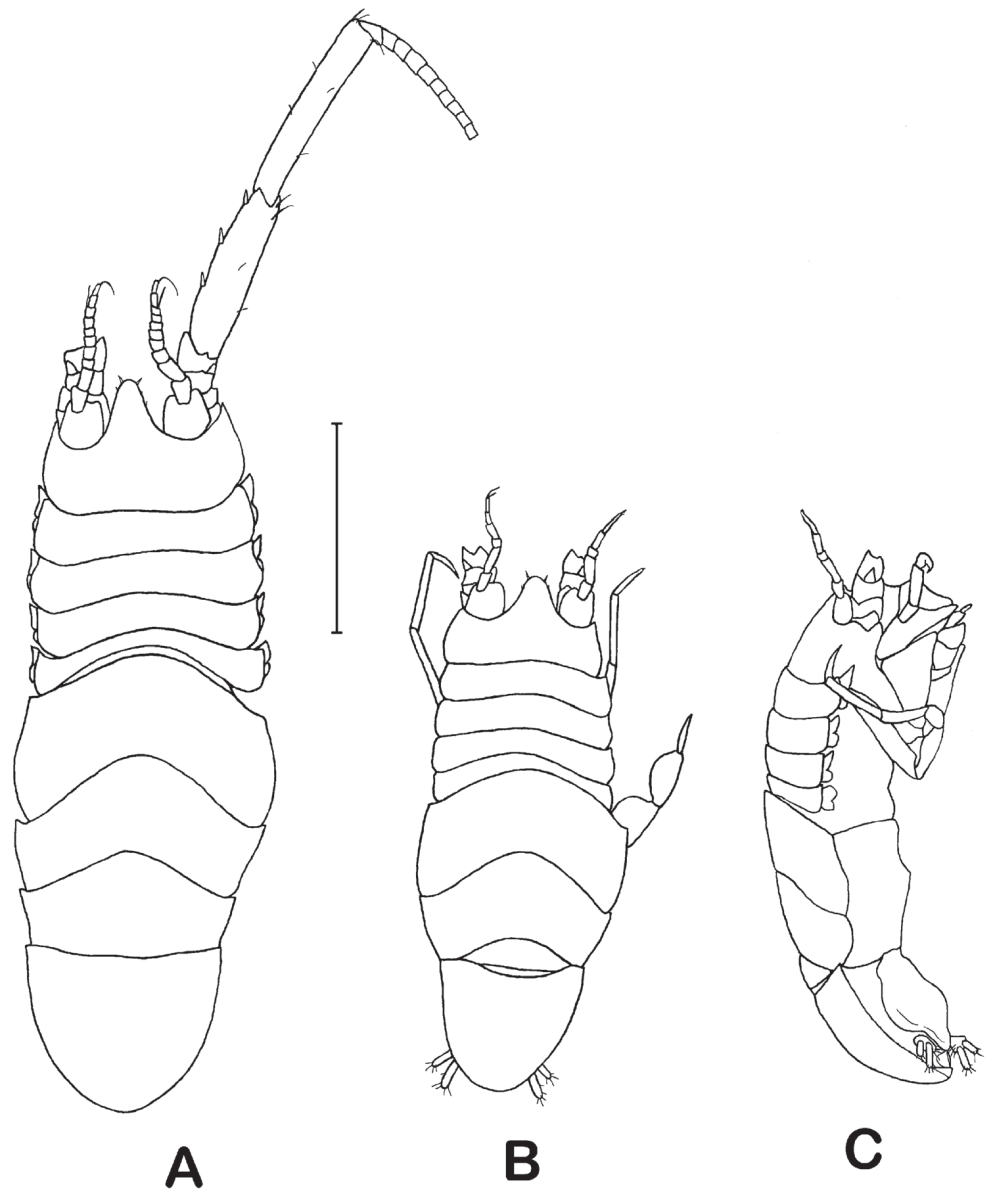
. *Dubinectes infirmus* sp. n. **A** paratype female (ZMH 42970) body dorsal view **B** immature female body dorsal view **C** body, lateral view.

**Figure 9.**
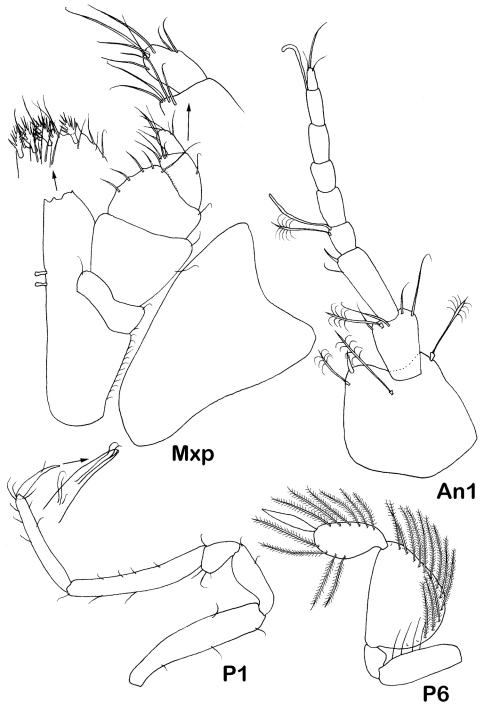
*Dubinectes infirmus* sp. n.Paratype immature female (ZMH 42970). Maxilliped with details, antennula, pereopod 1 and pereopod 6.

**Figure 10.**
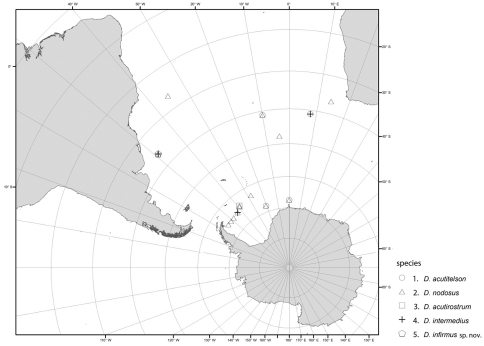
Distribution of species of the genus *Dubinectes*. **1** *Dubinectes acutitelson* **2** *Dubinectes nodosus* **3** *Dubinectes acutirostrum* **4** *Dubinectes intermedius* **5** *Dubinectes infirmus*

## Supplementary Material

XML Treatment for Dubinectes

XML Treatment for Dubinectes infirmus

XML Treatment for Dubinectes intermedius

XML Treatment for Dubinectes nodosus
